# Recent Advances in Black Phosphorous-Based Photocatalysts for Degradation of Emerging Contaminants

**DOI:** 10.3390/toxics11120982

**Published:** 2023-12-03

**Authors:** Zhaocheng Zhang, Dongyang He, Kangning Zhang, Hao Yang, Siyu Zhao, Jiao Qu

**Affiliations:** 1 Key Laboratory of Geographical Processes and Ecological Security of Changbai Mountains, Ministry of Education, School of Geographical Sciences, Northeast Normal University, Changchun 130024, China; zhangzc363@nenu.edu.cn; 2 School of Environment, Northeast Normal University, Changchun 130117, China; zhangkn924@nenu.edu.cn (K.Z.); yangh409@nenu.edu.cn (H.Y.); syzhao999@nenu.edu.cn (S.Z.)

**Keywords:** emerging contaminants, photocatalysis, black phosphorus, photocatalytic mechanism

## Abstract

The recalcitrant nature of emerging contaminants (ECs) in aquatic environments necessitates the development of effective strategies for their remediation, given the considerable impacts they pose on both human health and the delicate balance of the ecosystem. Semiconductor-based photocatalytic technology is recognized for its dual benefits in effectively addressing both ECs and energy-related challenges simultaneously. Among the plethora of photocatalysts, black phosphorus (BP) stands as a promising nonmetallic candidate, offering a host of advantages including its tunable direct band gap, broad-spectrum light absorption capabilities, and exceptional charge mobility. Nevertheless, pristine BP frequently underperforms, primarily due to issues related to its limited ambient stability and the rapid recombination of photogenerated electron–hole pairs. To overcome these challenges, substantial research efforts have been devoted to the creation of BP-based photocatalysts in recent years. However, there is a noticeable absence of reviews regarding the advancement of BP-based materials for the degradation of ECs in aqueous solutions. Therefore, to fill this gap, a comprehensive review is undertaken. In this review, we first present an in-depth examination of the fabrication processes for bulk BP and BP nanosheets (BPNS). The review conducts a thorough analysis and comparison of the merits and limitations inherent in each method, thereby delineating the most auspicious avenues for future research. Then, in line with the pathways followed by photogenerated electron–hole pairs at the interface, BP-based photocatalysts are systematically categorized into heterojunctions (Type I, Type II, Z-scheme, and S-scheme) and hybrids, and their photocatalytic performances against various ECs and the corresponding degradation mechanisms are comprehensively summarized. Finally, this review presents personal insights into the prospective avenues for advancing the field of BP-based photocatalysts for ECs remediation.

## 1. Introduction

Water, being a fundamental necessity for the survival of all living organisms, holds immense significance not only within the realm of biological systems but also within operations of numerous manufacturing industries [1,2,3]. In recent decades, a compelling realization has crystallized, unveiling the profound perils posed by water scarcity to the sustainable equilibrium of human society, primarily driven by escalating demands [4]. To meet the rising demands for water, it has become imperative to implement effective wastewater treatment and reutilization. The treatment process involves the reclamation of polluted water, originating from individuals or industrial processes, to attain an acceptable standard of quality. In the realm of water source recycling, the occurrence of trace organic pollutants in wastewater, their intricate interactions during treatment processes, and their consequential impacts on wastewater treatment and fresh water generation represent pivotal challenges [5]. As we delve into these challenges, it becomes evident that the unregulated release of novel pollutants, commonly referred to as emerging contaminants (ECs), has emerged as a pressing environmental concern [6,7,8,9].

In conventional wastewater treatment plants (WWTPs), the removal of ECs remains a significant challenge due to current technological limitations [10,11,12]. Detected at trace levels in liquid effluents, these compounds predominantly comprise pharmaceuticals, personal care products (PCPs), pesticides, steroid hormones, and a variety of other substances [13]. Among these, drugs such as antimicrobials, analgesics, anti-inflammatories, β-blockers, and lipid regulators, in conjunction with PCPs, are most prominent [14]. The occurrence of ECs in wastewater underscores pronounced concerns for aquatic ecosystem and human health, attributed to enduring exposure impacts on non-target species [15]. Certain pollutants under scrutiny function as endocrine-disrupting compounds (EDC), with potential implications for the modulation of hormone production, release, and activity in the organism [16]. Consequently, myriad health issues in animals may manifest, including breast or testicular cancer in humans. Furthermore, some studies have indicated that these pollutants can influence reproductive alterations in avian and aquatic species [17,18,19]. Guided by the precautionary principle, reusable water should be devoid of such compounds. As previously noted, numerous studies indicate that the technologies employed in WWTPs do not ensure effective removal of ECs, with concentrations ranging from ng/L to μg/L. Heterogeneous photocatalysis, an advanced oxidation process rooted in semiconductors capable of ultraviolet (UV)/visible light absorption, offers a cost-effective and adaptable approach for ECs removal [20,21,22,23]. When interfaced with water molecules, these catalysts induce the production of hydroxyl radicals (•OH) and superoxide ions (•O_2_^−^), acting as robust oxidants that can facilitate the degradation of ECs [24,25].

Recently, two-dimensional (2D) photocatalysts have garnered significant attention in the scientific and technological realms, surpassing interest in their traditional counterparts [26,27,28]. Following the pioneering isolation of thermodynamically stable monolayer graphene (GR) [29], there has been a surge of interest in exploring a plethora of other 2D photocatalysts, including but not limited to graphyne [30], graphdiyne [31], graphitic carbon nitride (g-C_3_N_4_) [32], transition metal chalcogenides (TMCs) [33], hexagonal boron nitride [34], and MXenes [35]. These 2D photocatalysts manifest remarkable physical and chemical characteristics, encompassing exceptional strengths, enormous surface-to-volume ratios, tunable band gaps, superior optical attributes, and enhanced electronic and thermal conductivities. In 2014, Zhang et al. reported the exemplary performance of black phosphorus (BP) transistors [36]. This material, once again, began to attract the attention of researchers. BP, a significant member of the phosphorus family, possesses notable advantages such as nontoxicity and enhanced thermodynamic stability at ambient temperatures, distinguishing it from white phosphorus (WP) and red phosphorus (RP). In the monolayer configuration of BP, each phosphorus atom forms covalent P–P bonds with three adjacent P atoms. These monolayer structures, featuring an interlayer spacing of approximately 5.5 Å, assemble into bulk BP through van der Waals interactions. BP emerges as a versatile material for diverse applications due to its high surface area and tunable electronic properties, making it an effective catalyst. Its high electrical conductivity, layer-dependent behavior, and anisotropic properties render it promising for energy storage applications. Additionally, BP, with its tunable band gap, strong light–matter interaction, and broadband absorption, stands out as a valuable candidate for optical devices. These multifaceted advantages position BP as a compelling material across catalyst, energy storage, and optics domains [37]. Subsequent studies have revealed numerous remarkable properties of BP that make it highly suitable as an emerging photocatalyst. BP is distinguished as a metal-free, direct band gap layered semiconductor [38]. Notably, its band gap can be adjusted from 0.3 to 2.0 eV contingent upon the layer’s thickness. This provides BP with strong optical absorption spanning from the UV to the near-infrared region [39]. Furthermore, BP exhibits a high charge mobility (approximately 1000 cm^2^ V^−1^ s^−1^) and a large surface area with efficient exposure of active sites [40]. Consequently, BP-based photocatalysts for energy conversion, water splitting, and environmental remediation, have garnered extensive attention in recent research [41,42,43,44,45,46]. Nevertheless, there is a limited number of reviews that focus on the utilization of BP in environmental remediation, specifically, its involvement in the photocatalytic degradation of ECs. In this review, we embark on a comprehensive exploration of BP and its potential as a versatile photocatalyst for addressing ECs. We commence with an in-depth analysis of the various fabrication methods for both bulk BP and BP nanosheets (BPNS), and assess the merits and limitations inherent in each of these fabrication techniques. Subsequently, we delve into the categorization of BP-based photocatalysts, systematically classifying them into heterojunctions (Type I, Type II, Z-scheme, and S-scheme) and hybrid materials. We thoroughly examine their photocatalytic performances in the degradation of various ECs, shedding light on the intricate degradation mechanisms. Lastly, we offer forward-looking perspectives on the future of BP-based photocatalysts in the realm of ECs remediation. This review serves as a valuable guide for researchers seeking innovative BP-based materials to tackle environmental challenges related to ECs.

## 2. Preparation of Bulk BP

For over a century, the synthesis of bulk BP crystals has seen continual advancements. These developments span a range of techniques, including high-temperature and high-pressure synthesis, mercury-catalyzed reaction, liquid bismuth recrystallization, ball milling method, and chemical vapor transport (CVT).

### 2.1. High-Temperature and High-Pressure Method

In 1914, Bridgman was the first to discover that white phosphorus (WP) could be converted into BP crystals in a remarkably short duration of 5 min when the reaction condition was elevated to 200 °C and 1.2 GPa [47]. Subsequently, Endo successfully synthesized BP single crystals with dimension exceeding 5 × 5 × 10 mm^3^, utilizing red phosphorus (RP) as the raw material. In a conventional high-pressure process at 1 GPa, polycrystalline BP was initially generated by heating RP to approximately 550 °C, followed by a melting process at around 900 °C. The synthesis apparatus was methodically cooled to a temperature of 600 °C at a decelerated rate of 0.5 °C/min, followed by the swift removal of the heating source to enable solidification and yield the ultimate BP crystals [48]. Nevertheless, the rigorous experimental conditions of the high-temperature and high-pressure method present safety hazards to researchers, and the deployment of this technique demands the utilization of energy-intensive instrumentation.

### 2.2. Mercury Catalysis and Liquid Bismuth Recrystallization Methods

To reduce the requisite pressure for synthesis, researchers have devised innovated approaches including mercury-catalyzed reactions and a liquid bismuth recrystallization technique. In 1955, Krebs et al. successfully achieved the thermal conversion of WP to BP under a low-pressure condition, utilizing a mercury catalyst [49]. However, the obtained BP crystals were contaminated with mercury, which posed the risk of leakage and subsequent long-term contamination of the BP crystals. Subsequently, Maruyama et al. successfully synthesized needle- and rod-shaped BP single crystals employing the liquid bismuth recrystallization technique [50]. Initially, commercial WP underwent purification via nitric acid treatment, followed by water–steam distillation. The purified WP was then co-located with bismuth in dual branches of a helium-sealed Pyrex glass tube. Subsequently, the molten bismuth was meticulously blended with WP, and the system was sustained at 400 °C for a duration of 20 h. Upon gradual cooling to ambient temperature, the solidified bismuth was dissolved in a 30% nitric acid solution, facilitating the extraction of BP from the remaining solution. However, these approaches to BP synthesis suffer from inherent drawbacks, including their toxicological implications and extended reaction durations. In combination with the unsatisfactory yield of BP, these limitations serve to restrict the long-term applicability of both methods.

### 2.3. Ball Milling Method

Ball milling serves as a mechanical approach for the synthesis of nanomaterials, wherein transient elevations in temperature and pressure, induced by the collisions between milling spheres and the vessel wall, facilitate phase transitions. Owing to the stringent conditions of high-temperature and high-pressure required for the transformation between RP and BP, ball milling has been adopted as a viable method for BP synthesis. Zhang et al. employed a high-energy ball milling method operating at 700 r/min for a duration of 2 h to synthesize BP using RP as the raw material [51]. In addition, BP powder was produced from RP via a high-energy mechanical milling procedure, utilizing a vibration mill by Shin et al. [52]. Approaches for the synthesis of BP through ball milling extend to high-energy planetary milling, high-energy shake milling, and plasma-assisted ball milling techniques. In a typical ball milling process, mechanical energy serves a critical function in facilitating the conversion of RP to BP, as elevated mechanical energy levels can expedite the grinding duration and enhance BP crystallinity [53]. However, characterization studies conducted by Zhang et al. in 2018 revealed that residual RP could persist within the BP product synthesized by the ball milling approach. Nevertheless, relative to the high-temperature and high-pressure approach, the ball milling method exhibits reduced energy consumption and yields a more favorable quantity of BP.

### 2.4. CVT Method

In 2007, Lange et al. were the first to achieve the synthesis of bulk BP single crystals utilizing CVT under a low-pressure condition [54]. Utilizing RP as the foundational material, this technique opted Au, Sn, and SnI_4_ as mineralizing agents, all of which were encapsulated in a vacuum-sealed quartz tube. The synthesis apparatus underwent a gradual heating process and was sustained at a temperature of 600 °C for a duration spanning 5 to 10 d. Subsequent to a gradual cooling process, bulk BP crystals were successfully acquired. In 2016, Wang et al. substantially enhanced the CVT technique by electing I_2_ as the mineralizing agent, thereby supplanting the use of SnI_4_ [55,56]. The fabrication cost of BP was markedly reduced through the substitution of inexpensive I_2_ for costly SnI_4_. Concurrently, specific metals or alloys provided a liquid medium in which I_2_ facilitated the migration of phosphorus during the reaction. Additionally, the entire preparation process was initially conducted under an Ar atmosphere, thereby streamlining the BP fabrication procedure. Leveraging these dual advantages, phosphorus was effectively solubilized at an elevated temperature and subsequently underwent controlled cooling to crystallize, culminating in the formation of BP crystals.

As elucidated in Table 1, the CVT method is identified as the most efficacious for BP synthesis, it also stands out as the most economically efficient in terms of resource consumption. On the other hand, the high-temperature and high-pressure method yields BP of the highest purity. Mercury catalysis and liquid bismuth recrystallization methods are toxic and produce various by-products. The ball milling method offers ease of operation, but it requires a long preparation time and results in the presence of other products. The stability and scalability of BP synthesis are effectively preserved through ball milling, conducted under an argon atmosphere, which safeguards the material from degradation. In contrast, alternative methods exhibit a certain degree of degradation when handling purified BP. Above all, the CVT method, acknowledged as the most commonly employed approach, has been optimized to yield the highest productivity and achieve complete separation of by-products through solid-phase extraction.

## 3. Preparation of BPNS

Usually, ultrathin 2D materials of atomic thickness exhibit a range of superior physicochemical properties relative to their bulk counterparts, including electronic anisotropy, planar conductivity, elevated surface activity, and tunable energy band structure. Consequently, the fabrication of atomically thin layers from layered bulk materials holds significant implications. In recent years, a plethora of methodologies for synthesizing ultrathin 2D BPNS have been advanced and documented. Up to the present, given both the frequency of methodological utilization and the quality of the resultant ultrathin 2D BPNS, techniques such as mechanical exfoliation, liquid-phase exfoliation, electrochemical expansion, and solvothermal method have gained wider application in the domain of BPNS synthesis.

### 3.1. Mechanical Exfoliation Method

Owing to the weak interlayer van der Waals interactions inherent in bulk BP crystals, mechanical force can readily facilitate their exfoliation into ultrathin 2D BPNS. In 2014, Liu et al. deployed a Scotch-tape-based microcleavage technique to achieve the exfoliation of layered bulk BP, culminating in the isolation of atomically thin single-layer BP [57]. Atomic force microscopy results revealed that the exfoliated BPNS displayed a step height of approximately 0.85 nm, indicative of the presence of single-layer BP. Both experimental and computational results affirmed that the band gap of BP was modulated by the number of layers. Although the mechanical exfoliation method can produce high-quality BPNS, the suboptimal production yield and the unavoidable contamination derived from residual adhesives remain significant drawbacks.

The ball-milling method serves as an alternative efficacious mechanical exfoliation technique for the synthesis of BPNS. Employing this method, Zhu et al. successfully generated BPNS by milling a mixture of anhydrous lithium hydroxide (LiOH) and bulk BP in an Ar atmosphere [58]. During the high-energy ball-milling procedure, the incorporation of LiOH as an additive serves a dual purposes: it inhibited the phase transformation of BP into RP and facilitated hydroxyl functionalization at the edges of the BPNS, thereby augmenting their environmental stability. Microscopic characterizations indicated that the resultant BPNS exhibited dimensions ranging from 300 to 500 nm and thicknesses between 0.7 and 6.0 nm. Consequently, in the absence of any noble metal cocatalysts, these BPNS demonstrated a photocatalytic H_2_ production rate approximately 18-fold greater than that of bulk BP under visible-light exposure. Despite the rapid synthesis afforded by the ball-milling technique, the facile oxidative degradation of the exfoliated BPNS upon exposure to ambient conditions continues to pose a significant challenge.

### 3.2. Liquid-Phase Exfoliation Method

In recent years, the liquid-phase exfoliation technique has gained widespread adoption for the synthesis of various 2D nanosheet materials [59,60,61]. Typically, layered bulk materials are dispersed in solvents specifically chosen to match their surface energy. Subsequently, ultrasonic oscillation facilitates the detachment of a 2D nanosheet from the parent bulk crystal. Moreover, the dimension and thickness of the resulting 2D nanosheet can be fine-tuned through the judicious selection of solvents and the optimization of exfoliation–centrifugation parameters, such as duration, temperature, and centrifugal velocity. In 2014, Brent et al. were the first to achieve the exfoliation of bulk BP in *N*-methyl-2-pyrrolidone (NMP) solution, strategically modulating the exfoliation time to obtain few-layer BPNS [62]. Following 24 h of exfoliation, the resulting BPNS predominantly comprised three- to five-layer phosphorene structures, with dimensions of 200 × 200 nm and thicknesses ranging from 3.5 to 5.0 nm. Extending the exfoliation duration to 48 h led to the decomposition of larger flakes into smaller BPNS, characterized by dimensions of 20 × 20 nm and thicknesses between 0.9 and 1.6 nm, primarily consisting of single and bilayer phosphorene. Building upon this preparative approach, Guo and colleagues employed a saturated NaOH NMP solution to augment the yield of thin-layer phosphorene [63]. The resultant phosphorene demonstrated enduring stability in both NMP and aqueous environments. Concurrently, the researchers discovered that by adjusting the centrifugal speed, they were able to obtain phosphorene with a relatively uniform size and thickness.

Moreover, the choice of appropriate solvent is crucial for the successful exfoliation and high-yield production of 2D BPNS. For instance, Lin et al. employed a range of solvents including ethanol, isopropyl alcohol (IPA), acetone, and NMP to investigate the exfoliation outcomes under identical experimental conditions [64]. Despite the uniformity in size (~100 nm) and thickness (2–7 nm) of the BPNS acquired through ultrasonic treatment in diverse solvents, the final yield varied significantly among the solvents, with IPA yielding the highest output. Zhang et al. evaluated the exfoliation efficacy of bulk BP in an array of solvents with varying surface tensions, such as NMP, *N*,*N*-dimethylformamide (DMF), dimethyl sulfoxide (DMSO), formamide, tetrahydrofuran (THF), IPA, ethanol, methanol, acetone, and deionized water [65]. The concentrations of the resulting few-layer BP varied across solvents, exhibiting the following hierarchy: formamide > DMSO > DMF/NMP/IPA > ethanol/methanol > acetone/THF/deionized water. Additionally, the high-quality BPNS demonstrated remarkable stability in formamide, enduring for hundreds of hours without discernible degradation.

Consequently, due to its cost-effectiveness and controllable procedures, the liquid-phase exfoliation technique serves as a robust platform for the preparation of 2D BPNS materials. Presently, a multitude of photocatalytic investigations involving 2D ultrathin BPNS utilize this liquid-phase exfoliation method.

### 3.3. Electrochemical Electrode Stripping Method

Electrochemical exfoliation stands out as a notably efficient and controllable methodology. This technique affords the production of BPNS with meticulous regulation of size and thickness, achieved by judiciously applying distinct voltages over varying durations. The process bifurcates into anode and cathode exfoliation, contingent upon the specific locale of the exfoliating materials. In 2015, Erande and co-workers pioneered the utilization of anode exfoliation for the synthesis of BPNS. Employing a positive bias voltage of +7 V and a current of approximately 0.2 A on the working electrode for a duration of 50 min, BPNS manifested nanosheet-like characteristics, featuring lateral dimensions spanning 5 to 10 mm and a thickness measuring around 1.4 nm. In contrast to anode exfoliation, cathode exfoliation circumvents the generation of oxygen-containing free radicals, resulting in phosphorene products devoid of visible surface defects or oxidative damage. Mayorga-Martinez et al. reported a singular, solution-based electrochemical exfoliation employing bipolar electrodes. This approach facilitates the exfoliation and downsizing of layered BP microparticles into nanoparticles, resulting in heightened electrocatalytic activity for the hydrogen evolution reaction [66]. What is more, Baboukani et al. demonstrated a singular, facile, and eco-friendly approach for the one-step bipolar electrochemical exfoliation and deposition of BPNS onto a positively biased feeding electrode [67]. The exfoliated and deposited material exhibits a high-quality phosphorene with an orthorhombic crystal structure, firmly adhering to the substrate with a fractal and structured morphology. The electrochemical assessment of the material was conducted using a symmetric two-electrode configuration immersed in an aqueous electrolyte for capacitive energy storage applications. The findings unveiled fractional-order capacitive behavior in a low-to-medium frequency range, coupled with exceptional stability and reversibility sustained over a remarkable 40,000 cycles. As previously indicated, cathodic exfoliation involves the application of a negative voltage to bulk BP, driving cation insertion from the electrolyte. The expansion force generated by H_2_ is harnessed to promote BP exfoliation. In contrast to anodic exfoliation, cathodic exfoliation excluded the generation of oxygen-containing free radicals, resulting in phosphorene devoid of surface defects and functional groups.

Anodic, cathodic, and bipolar exfoliation traditionally relied on bulk BP crystals, which, due to their elevated cost, posed a hindrance to the widespread application of electrochemical exfoliation for BPNS preparation. The utilization of BP powder as a raw material presented a viable solution to alleviate this economic constraint. In 2018, Xiao et al. positioned BP powder within a porous plastic tube, employing one platinum plate as the cathode and another as the anode [68]. Subsequently, both electrodes were submerged in a solution of propylene carbonate infused with tetra-n-butyl ammonium hydrogen sulfate (–TBA•HSO_4_), and a voltage of 30 V was applied for a duration of 12 h. Following the electrochemical expansion phase, the expanded powder was subjected to ultrasonic cleaning in a pure propylene carbonate (PC) solution for 0.5 h. It was then filtered and rinsed thrice before being vacuum-dried at 60 °C for a period of 8 h to yield the BPNS. Wang et al. similarly accomplished the electrochemical stripping of BP using TBA•HSO_4_, a solution noted for its high electrochemical stripping efficiency [69]. Comparative studies with TBA•ClO_4_ and TBA•PF6 have demonstrated that both negative hydrogen ions (H^−^) and sulfate ions (SO_4_^2−^) play equally significant roles in the electrochemical stripping process [70,71,72]. Polyurethane acrylate and tetraallyl ammonium salt also have been employed as electrolytes, demonstrating commendable efficacy in material preparation [73,74]. Huang et al. introduced electrochemical cathodic stripping as a method for synthesizing BPNS, revealing that the number of BP layers could be controlled through the manipulation of the applied electrical potential [75].

In comparison to other preparation approaches, the electrochemical stripping method provided a highly efficient and convenient means for generating BPNS, achieving gram-scale production within a span of 1 h. The dimensions of the material extended to several tens of microns, facilitating rapid exfoliation and minimizing the potential for degradation and fragmentation induced by water and ultrasonic treatment. Additionally, the electrochemical cathodic stripping process effectively precluded oxidation, thereby yielding high-quality BPNS.

### 3.4. Chemical Vapor Deposition Method

Chemical vapor deposition (CVD) is a widely employed methodology for the preparation of 2D materials [76]. Using a two-step heating CVT approach, Zhang et al. streamlined the isolation and purification of orthogonal BP single crystals. Notably, unparalleled hole rate and electron mobility were exhibited by the BPNS derived via CVD, setting a new benchmark among BPNS synthesized by various methods [77]. Smith et al. introduced an in situ CVD technique that enabled the growth of large-area BPNS [78]. Intriguingly, RP could serve as a direct precursor for BPNS synthesis via this CVD approach. Relative to alternative approaches, CVD offers a cost-effective synthesis route. Despite its economic and flexible nature, the method suffers from low yield and product impurity. While current limitations exist, CVD holds promise for economical production in the future.

### 3.5. Pulsed Laser Method

Pulsed laser deposition employs laser irradiation to ablate a target, subsequently depositing the ejected plasma onto a substrate for thin-film formation [79]. Remarkably, this technique is universally applicable across a broad spectrum of materials for thin-film synthesis. Yang et al. adeptly achieved the deposition of BPNS at 150 °C via pulsed laser deposition [70]. Concurrently, Li et al. transformed RP into BP using a pulse laser approach at conditions of 700 °C and 1.5 GPa, resulting in BPNS formation on sapphire substrates [80]. Intriguingly, the pulsed laser technique allows for modulation of film thickness through adjustment of the pulse count, thereby enabling the production of BPNS with uniform specifications. The pulsed laser technique yields BPNS with greater precision compared to prior methods. Additionally, this approach offers the flexibility to tailor the thickness of BPNS as needed.

A comparative analysis of various BPNS preparation methods, as delineated in Table 2, reveals that liquid-phase exfoliation remains prevalent in current applications. However, the pulsed laser emerges as a particularly promising strategy owing to its tunable preparation parameters. The CVD approach, offering time-efficient synthesis of BPNS, holds significant promise for future research endeavors. Nonetheless, the current CVD methodology requires refinement, as impurities persist in the resultant BPNS. The mechanical exfoliation method and pulsed laser method can yield high-quality thin layers of BP; however, their drawbacks include low production yields, expensive requirements and the inability to ensure complete isolation of few-layer BP from the external environment. In contrast to alternative methods, liquid-phase exfoliation employed solvents to create a barrier, effectively isolating BPNS from external factors, especially oxygen and water, thereby ensuring the stability and scalability of BPNS.

## 4. Photocatalytic Degradation of ECs Using BP-Based Materials

### 4.1. Heterojunctions

Typically, the design and assembly of heterojunction photocatalysts, comprising distinct semiconductors, emerge as a potent strategy to achieve superior photocatalytic performance. Recently, the development and fabrication of BP-based heterojunction photocatalysts have surged, driven by three primary advantages of BP [81]. Firstly, constructing a heterojunction interface between BP and other materials facilitates the effective spatial separation and migration of photogenerated electrons (e^−^) and holes (h^+^). Secondly, considering the light absorption characteristic of photocatalysts, BP-based heterojunctions can efficiently capitalize on the solar spectrum due to the extensive absorption range of BP that spans from a UV to a near-infrared region. Thirdly, given its attributes such as a tunable band gap, a large specific surface area (when in the form of BPNS), and excellent charge mobility, BP emerges as a metal-free cocatalyst, bolstering the surface redox kinetics in heterojunction photocatalysts. Based on the varied e^−^–h^+^ pair separation and migration pathways observed in prior heterojunction systems involving BP and its paired photocatalysts, the photocatalytic degradation of ECs using BP-based heterojunctions can be classified into four distinct categories: (1) Type I, (2) Type II, (3) Z-scheme, (4) S-scheme alignments.

#### 4.1.1. Type I Heterojunction

**Figure 1:**
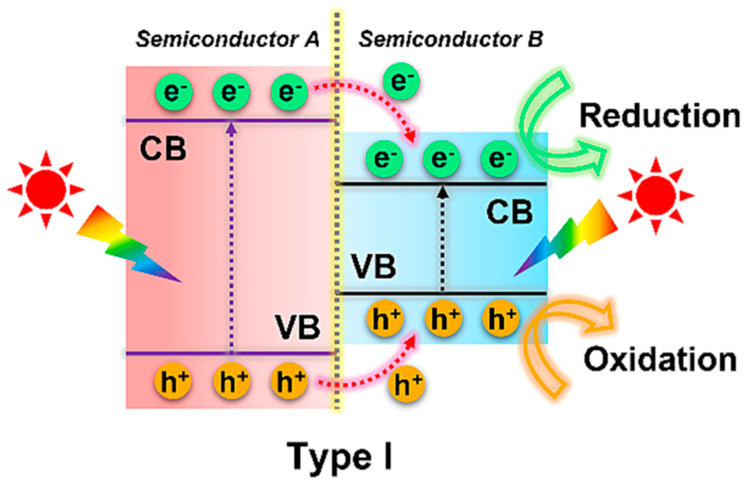
Schematic illustrating the separation and migration of interface charge carriers under light irradiation in Type I heterojunction system. Copyright Year 2023, *Separation and Purification Technology* © Elsevier Pvt Ltd., Amsterdam, The Netherlands.

In a typical Type I heterojunction photocatalyst (Figure 1), the conduction band (CB) and valence band (VB) of semiconductor A situate above and below the corresponding bands of semiconductor B, respectively. Upon light irradiation, e^−^ and h^+^ originating from semiconductor A transition to the CB and VB of semiconductor B, respectively.

**Figure 2:**
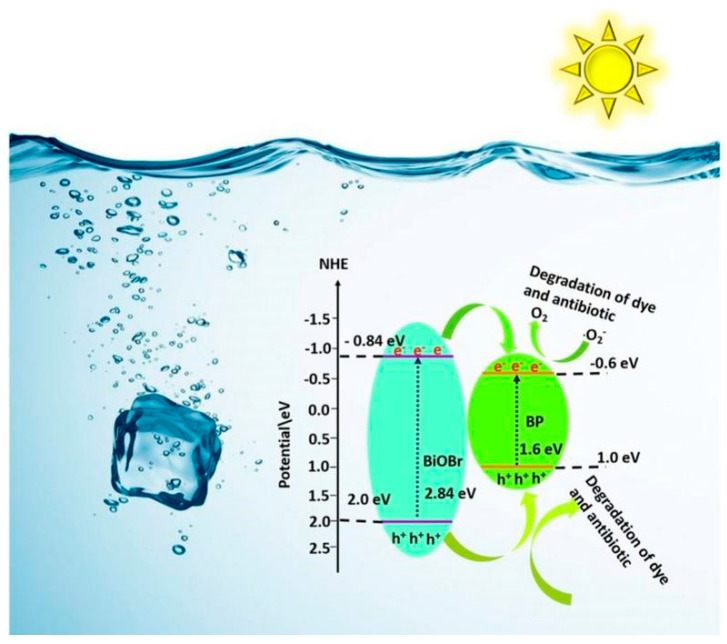
Diagrams for energy levels and band structures of BiOBr and BPNS. Copyright Year 2021, *Colloids and Surfaces A: Physicochemical and Engineering Aspects* © Elsevier Pvt Ltd., Amsterdam, The Netherlands.

Li et al. employed a one-step co-precipitation approach to combine poly dimethyldiallyl ammonium chloride (PDDA)-passivated BPNS with BiOBr, resulting in the formation of BPNS–BiOBr heterojunctions [82]. Regarding the BPNS–BiOBr samples, their UV and visible-light absorption capabilities exhibited enhancement with the BPNS content increased, accompanied by a redshift in absorption edges. This outcome suggested that the introduction of BPNS extended the visible-light utilization of the photocatalytic system. Such enhancements were highly beneficial for improving the photocatalytic efficiency of the catalyst, leading to augmented photocatalytic activity and enhanced generation of photoinduced carriers. Hence, the BPNS–BiOBr heterojunctions demonstrated exceptional photocatalytic performance, achieving a remarkable 98.2% degradation efficiency for ciprofloxacin (CIP) within 60 min. The augmented photocatalytic activity observed in the heterojunctions could be ascribed to the successful establishment of a Type I bandgap configuration (Figure 2). Notably, the passivating influence of PDDA enabled the heterojunctions to uphold remarkable photostability, even through multiple degradation cycles.

**Figure 3:**
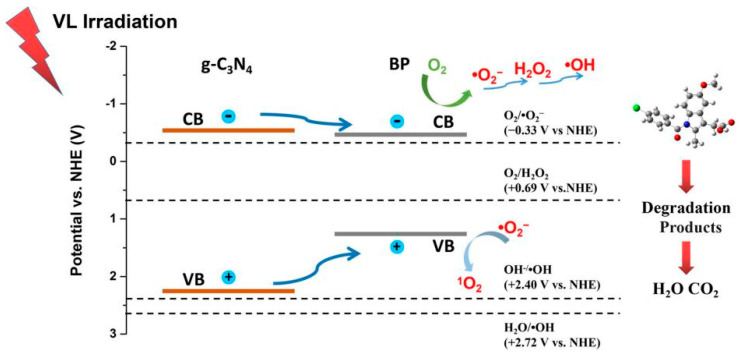
Proposed photocatalytic reaction mechanism of 8%BP-g-C_3_N_4_. Copyright Year 2022, *Science of the Total Environment* © Elsevier Pvt Ltd., Amsterdam, The Netherlands.

With its aptly positioned CB and VB, g-C_3_N_4_ can readily form a Type I heterojunction photocatalyst in conjunction with BP [83]. He and colleagues fabricated a highly active, metal-free photocatalyst in the form of a BP-g-C_3_N_4_ heterostructure using an innovative exfoliation approach [84]. The 8%BP-g-C_3_N_4_ heterostructure demonstrated a significantly enhanced photocatalytic efficiency in the decomposition of indomethacin (IDM) when compared to pure g-C_3_N_4_, achieving a remarkable 99.2% removal of IDM within 30 min of visible-light irradiation. The synergy between g-C_3_N_4_ and BP effectively enhanced the transfer and separation of charge carriers. This phenomenon could be elucidated by the effective establishment of a Type I heterojunction at the interfaces between g-C_3_N_4_ and BP. In this configuration, efficient separation of charge carriers ensued as a consequence of the disparities in the migration rates between e^−^ and h^+^ traveling from g-C_3_N_4_ to BP. This capability enabled the 8% BP-g-C_3_N_4_ to efficiently generate reactive oxygen species (ROS) for the decomposition of IDM. By employing quantitative structure–activity relationships (QSARs) via the ECOSAR program, it was determined that the predicted toxicity of all recognized intermediates was lower than that of IDM itself. Additionally, the majority of the generated byproducts could be characterized as non-harmful. Through four consecutive cycles of photocatalytic IDM degradation, 8%BP-g-C_3_N_4_ demonstrated stability and reusability, highlighting its potential as a stable and recyclable catalyst. Meanwhile, Jin et al. effectively engineered a Type I heterojunction, incorporating BP and g-C_3_N_4_, for utilization in the photocatalytic treatment of tetracycline hydrochloride (HTC) [85]. Under simulated visible-light irradiation, the 6%BP/g-C_3_N_4_ (optimized sample) exhibited a degradation efficiency of up to 99% within 30 min for HTC. Furthermore, under real sunlight illumination, the degradation efficiency of HTC achieved by 6%BP/g-C_3_N_4_ was 2.7 times greater than that of the commonly employed P25 TiO_2_. The experimental results demonstrated that the introduction of BP onto g-C_3_N_4_ facilitated charge carrier separation and enhanced light absorption in the visible-light range, thereby significantly boosting the catalytic activity. For HTC degradation as shown in Figure 3, the photoexcited e^−^ generated on BP engaged in the reaction with O_2_, leading to the production of •O_2_^−^, driven by the more negative CB potential of BP compared to the redox potential of O_2_/•O_2_^−^ (approximately −0.33 V versus NHE). It was essential to emphasize that the standard redox potentials of OH^−^/•OH (1.99 V versus NHE) and H_2_O/•OH (2.37 V versus NHE) were more positive than the VB potential of BP, signifying that the direct generation of •OH was thermodynamically impractical. As a result, •OH was formed through a subsequent reaction of •O_2_^−^ with e^−^. Additionally, •O_2_^−^ could further react with photogenerated h^+^ to yield ^1^O_2_. Furthermore, O_2_ could react with e^−^ to produce H_2_O_2_ via the two-step single-electron and the one-step two-electron reduction reactions. Ultimately, the generated ROS participated in the degradation of HTC.

Within BP-based Type I heterojunction photocatalysts, the synergistic effects of robust interfacial contact and chemical bonding, coupled with the extensive light absorption characteristic of BP, significantly enhances the photocatalytic performance. Nonetheless, a limitation arises as both photogenerated e^−^ and h^+^ accumulate on the same semiconductor, thereby resulting in inadequate separation of the photogenerated carriers. Consequently, it becomes imperative to establish an effective mechanism for the spatial separation and migration of photogenerated carriers in the development of BP-based heterojunction photocatalysts.

#### 4.1.2. Type II Heterojunction

**Figure 4:**
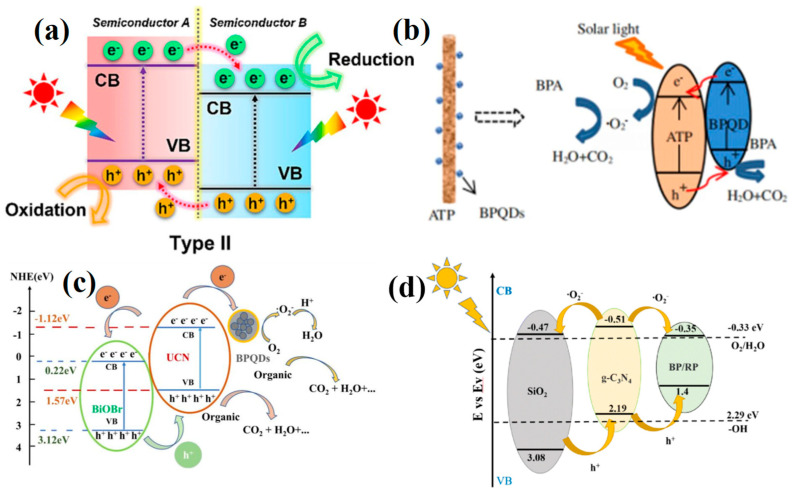
(**a**) Schematic illustrating the separation and migration of interface charge carriers under light irradiation in Type II heterojunction system. Copyright Year 2023, *Separation and Purification Technology* © Elsevier Pvt Ltd., Amsterdam, The Netherlands. (**b**) Photocatalytic degradation mechanism of BPQDs/ATP. Copyright Year 2017, *Functional Materials Letters* © World Scientific Publishing Company, Singapore. (**c**) The band structure diagram of tetracycline degradation and possible mechanisms. Copyright Year 2018, *Polymers* © Multidisciplinary Digital Publishing Institute, Basel, Switzerland. (**d**) The photocatalytic mechanism of BP/RP-g-C_3_N_4_/SiO_2_. Copyright Year 2023, *RSC Advances* © Royal Society of Chemistry, London, UK.

Type II heterojunction photocatalysts typically involve a pair of semiconductors possessing a distinct staggered energy band configuration. In this arrangement, the CB and VB levels of semiconductor A surpass those of semiconductor B, as depicted in Figure 4a. Consequently, throughout the course of photocatalysis, the photogenerated e^−^ within the CB of semiconductor A migrate to the CB of semiconductor B. Simultaneously, the photogenerated h^+^ within the CB of semiconductor B transfer to the VB of semiconductor A, facilitating an efficient spatial separation of e^−^–h^+^ pairs.

For instance, Li et al. utilized a facile hydrothermal deposition method to fabricate BPQDs/attapulgite (ATP) nanocomposites. UV-vis analysis unveiled that the BPQDs/ATP composite exhibited a broader range of visible-light absorption in contrast to pristine ATP [86]. The assessment of photocatalytic efficacy was conducted by examining the degradation of bisphenol A (BPA), wherein the findings revealed that, under 180 min of solar light irradiation, BPQDs/ATP achieved an impressive degradation rate of 90%. This heightened photocatalytic performance could be attributed to the synergistic effect of the coherent heterostructure created by BPQDs and ATP. This synergy arose from the sensitization effect induced by BPQDs and the enhanced facilitation of charge separation. The Type II heterostructure formed by ATP and BPQDs amplified solar light utilization and expedited the separation of e^−^ and h^+^ (Figure 4b). This concerted effect significantly augmented the photocatalytic activity of BPQDs/ATP.

Furthermore, several ternary BP-based heterojunction materials have also been synthesized for photocatalytic degradation of ECs. Jiang et al. fabricated ternary heterostructures of BiOBr/ultrathin g-C_3_N_4_(UCN), which were modified with BPQDs, through a straightforward procedure involving water bath heating and sonication [87]. This ternary heterostructure was subsequently employed for the photocatalytic degradation of tetracycline (TC) under visible light, achieving an impressive efficiency of up to 92% after 3 h of irradiation. Consequently, the photodegradation efficiency exhibited a significant enhancement in comparison to that of individual UCN, BiOBr, and BPQDs. The fabricated ternary heterostructure enhanced the charge separation efficiency, thereby resulting in an augmented photodegradation efficiency. A schematic diagram in Figure 4c illustrated the band structure diagram of the ternary heterostructure and the potential mechanisms involved for TC degradation. The CB and VB of BiOBr were determined to be 0.22 and 3.12 V, respectively, while those of UCN were −1.12 and 1.57 V, respectively. Due to the particle size relationship among the BPQDs, the band gap fell within the range of 0.3 to 1.8 V, with the specific value not provided here. Nevertheless, it was evident that this ternary heterogeneous structure established a bandgap conducive to the efficient generation and separation of photogenerated carriers, thereby significantly enhancing the role of •O_2_^−^ and h^+^ in catalytic TC degradation. To evaluate the stability of the BiOBr/UCN/BPQDs, the multiple cycles of photocatalytic TC degradation and X-ray diffraction (XRD) analysis were conducted. The catalytic performance of BiOBr/UCN/BPQDs showed only marginal decline, indicating the excellent structural stability of the material.

Recently, a novel heterojunction material, BP/RP-g-C_3_N_4_/SiO_2_, was successfully synthesized through a one-step ball milling process [88]. Subsequently, its photocatalytic performance was investigated by assessing the degradation of ofloxacin (OFL) under simulated sunlight. The development of an in situ BP/RP heterojunction resulted in the establishment of a high-efficiency interface interaction between distinct semiconductors, thereby facilitating the effective separation of photogenerated carriers. Comprehensive material characterization confirmed the successful creation of a multi-heterogeneous structure. Moreover, a Type II heterojunction emerged at the g-C_3_N_4_ and BP/RP interface, assuming a pivotal role in the degradation process while enhancing electron transfer (Figure 4d). The degradation of OFL achieved an 85.3% removal rate after 50 min of illumination when using BP/RP-g-C_3_N_4_/SiO_2_, whereas g-C_3_N_4_/SiO_2_ resulted in a significantly lower degradation rate of only 35.4%. Further discussions regarding the mechanisms revealed that •O_2_^−^ and h^+^ were identified as the primary active substances responsible for degrading OFL. In addition, after four degradation cycles, BP/RP-g-C_3_N_4_/SiO_2_ achieved a 76% degradation of OFL, with no significant alterations observed in the Raman test results, suggesting robust stability of the material.

These studies mentioned above suggest that the fabrication of BP-based Type II heterojunction photocatalysts effectively facilitates the separation and migration of photogenerated carriers. Nevertheless, it is noteworthy that the redox reactions take place at relatively low reduction and oxidation potentials of the semiconductors. Therefore, aside from achieving efficient e^−^–h^+^ separation, it is imperative to maintain a higher redox capacity when constructing BP-based heterojunction photocatalytic materials.

#### 4.1.3. Z-Scheme Heterojunction

**Figure 5:**
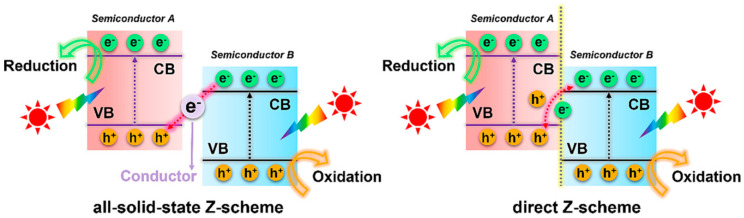
Schematic illustrating the separation and migration of interface charge carriers under light irradiation in Z-scheme heterojunction system. Copyright Year 2023, *Separation and Purification Technology* © Elsevier Pvt Ltd., Amsterdam, The Netherlands.

When compared to conventional Type I and Type II heterojunction systems, a Z-scheme heterojunction system can maximize the redox capacity of the heterojunction catalyst by enhancing the spatial separation of interfacial e^−^–h^+^ pairs. As depicted in Figure 5, an all-solid-state Z-scheme photocatalyst comprises two distinct semiconductors and an electron mediator. Upon exposure to light, e^−^ located in the VB of semiconductor B initially undergo excitation to reach the CB. Subsequently, these e^−^ transfer to the VB of semiconductor A with the assistance of an electron mediator, where they recombine with h^+^. This process results in spatial separation of e^−^–h^+^ pairs and the attainment of a high redox potential.

**Figure 6:**
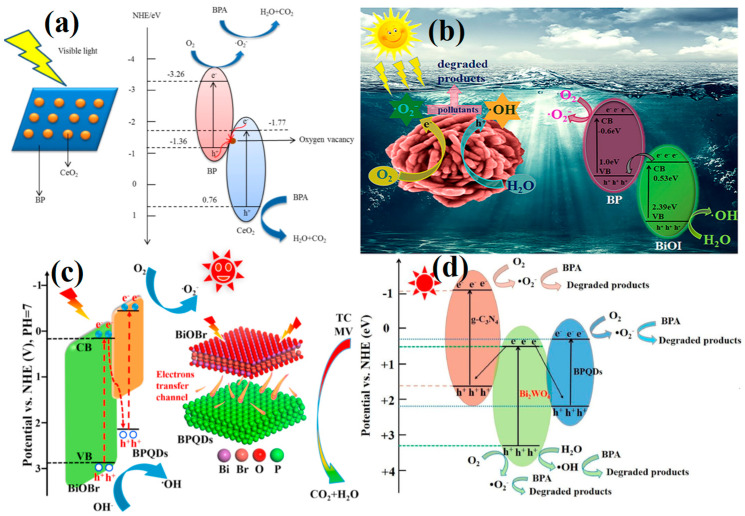
(**a**) Photocatalytic degradation mechanism by BP/CeO_2_ composite. Copyright Year 2018, *Journal of Materials Science: Materials in Electronics* © Springer, Berlin, Germany. (**b**) Photocatalytic mechanism of pollutants degradation by F-BP/BiOI heterojunction under visible-light irradiation. Copyright Year 2020, *Journal of Colloid and Interface Science* © Elsevier Pvt Ltd., Amsterdam, The Netherlands. (**c**) Photocatalytic schematic illustration for BPQDs/BiOBr thin film sample under visible light irradiation. Copyright Year 2021, *Journal of Materiomics* © Elsevier Pvt Ltd., Amsterdam, The Netherlands. (**d**) Possible mechanism of BPA degradation by Bi_2_WO_6_/g-C_3_N_4_/BPQDs composites. Copyright Year 2023, *Journal of Environmental Sciences* © Elsevier Pvt Ltd., Amsterdam, The Netherlands.

In 2018, CeO_2_/BP composites were prepared using a two-step hydrothermal and deposition method [89]. This process resulted in the uniform coating of CeO_2_ nanoparticles onto BP sheets, with a minor incorporation of BP into a CeO_2_ crystal lattice, leading to the creation of oxygen vacancies within the CeO_2_ structure. Close interaction between BP and CeO_2_ was established through Ce–O–P bonds. When employed as a photocatalyst, the CeO_2_/BP composite achieved an impressive 82.3% degradation rate of BPA within 180 min. This performance surpassed that of individual BP and CeO_2_ photocatalysts. A synergistic action of •O_2_^−^ and h^+^ was observed to mineralize BPA into H_2_O and CO_2_, as evidenced by scavenger experiments. The enhancement in photocatalytic activity was primarily attributed to the formation of an indirect Z-scheme CeO_2_/BP heterojunction, facilitated by oxygen vacancies (Figure 6a). This heterojunction effectively separated e^−^–h^+^ pairs while maintaining high VB and CB potentials, thereby improving the photocatalytic degradation capability.

Bismuth-based oxide materials, characterized by their distinctive electronic structure, well-suited bandgap dimension, facile synthesis, cost-effectiveness, and readily available source materials, have garnered substantial interest in recent years [90,91]. They have been widely employed in the field of photocatalysis and exhibit substantial potential for various applications. In 2020, Jing and colleagues presented a novel visible-light-responsive photocatalyst consisting of PDDA-functionalized BP (F-BP) and BiOI with a direct Z-scheme alignment (Figure 6b) [92]. This photocatalyst exhibited remarkable efficiency in the degradation of methylene blue (MB) and TC. The direct Z-scheme heterojunction significantly enhanced the separation and transfer rates of e^−^–h^+^ between BP and BiOI, preserving the high redox capacity of the F-BP/BiOI heterojunction photocatalyst. As a result, the accumulated e^−^ in the CB of BP exhibited substantial reducing power, effectively generating •O_2_^−^ from O_2_, while the h^+^ in the VB of BiOI participated in reactions with H_2_O to form •OH. These newly generated ROS, characterized by strong oxidative capabilities, further oxidized pollutants. Consequently, the optimized F-BP/BiOI heterojunction photocatalyst displayed an outstanding photocatalytic performance, achieving removal efficiencies of 97.6% for MB and 90.0% for TC. In addition, the photocatalyst exhibits commendable stability even after three catalytic cycles. Furthermore, a direct Z-scheme photocatalytic system was successfully engineered by Yao et al. through the integration of BPQDs onto the surface of BiOBr thin film [93]. The optimized BPQDs/BiOBr-1 thin film exhibited a significantly enhanced photocatalytic activity for the degradation of methyl violet (MV) and TC, with efficiencies 3 and 3.65 times higher, respectively, compared to its pure BiOBr counterpart. Radical trapping experiments have determined that the active species involved in the reaction were h^+^, •O_2_^−^, and •OH. The formation of a direct Z-scheme photocatalytic system leaded to e^−^ accumulation in the CB of BPQDs and h^+^ accumulation in the VB of BiOBr, promoting the generation of h^+^ and •O_2_^−^, thus accelerating the photocatalytic reaction (Figure 6c). Additionally, the enhanced efficiency of charge separation, improved visible-light absorption, and increased specific surface area also contributed to the enhanced photocatalytic performance of BPQDs/BiOBr. In addition, Du and colleagues developed a novel composite involving dual Z-scheme heterojunctions, combining Bi_2_WO_6_, g-C_3_N_4_, and BPQDs [94]. The Bi_2_WO_6_/g-C_3_N_4_/BPQDs was applied for the photocatalytic degradation of BPA under visible-light irradiation. By optimizing the mass ratio of g-C_3_N_4_ and BPQDs within the Bi_2_WO_6_/g-C_3_N_4_/BPQDs composites, the visible-light harvesting capacity could be significantly enhanced. This optimization leaded to improved charge separation efficiency. Consequently, the photocatalytic activity for BPA degradation was greatly enhanced, achieving an impressive degradation rate of 95.6% within 120 min for a 20 mg/L BPA solution. This performance surpassed that of individual components such as Bi_2_WO_6_ (63.7%), g-C_3_N_4_ (25.0%), BPQDs (8.5%), and Bi_2_WO_6_/g-C_3_N_4_ (79.6%) composite, highlighting the superiority of the Bi_2_WO_6_/g-C_3_N_4_/BPQDs composite system. Radical trapping experiments provided crucial insights, revealing that photogenerated h^+^ and •O_2_^−^ played pivotal roles in the photocatalytic degradation of BPA (Figure 6d).

**Figure 7:**
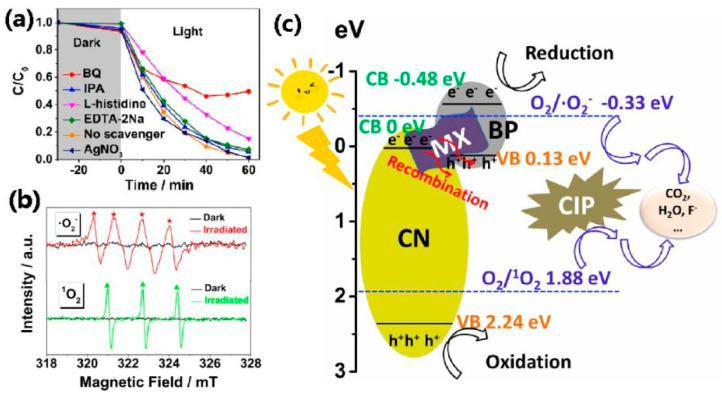
(**a**) Degradation (C/C_0_ plot) of CIP in photocatalysis, BQ, IPA, l-histidine, EDTA-2Na, and AgNO_3_ as scavengers, (**b**) ESR spectra of •O_2_^−^ and ^1^O_2_, (**c**) band structure of the C_0.2_X_0.01_B_0.01_ samples for CIP photodegradation under visible light irradiation. Copyright Year 2021, *Applied Catalysis B: Environmental* © Elsevier Pvt Ltd., Amsterdam, The Netherlands.

Recent investigations have confirmed that the high-conductivity MXene can serve as an effective electron mediator, facilitating the rapid transfer of photogenerated e^−^ from semiconductors to MXene. This property has found broad applications in the realm of heterojunction photocatalysts [95,96,97,98,99]. Zhou et al. created a novel and highly efficient dual-effective Z-scheme heterojunction photocatalyst, denoted as g-C_3_N_4_/Ti_3_C_2_ MXene/BP (CXB), through a meticulously designed calcination process [100]. This photocatalyst achieved exceptional photodegradation efficiency, exceeding 99%, for CIP under visible-light irradiation (λ > 420 nm) within a mere 60 min. Moreover, additional investigations, as depicted in Figure 7a (quenching experiments) and Figure 7b (electron spin resonance spectra), served to corroborate that the primary oxidative species responsible for the photodegradation reaction were •O_2_^−^ and ^1^O_2_. In Figure 7c, the carrier transfer pathway within the CXB heterojunction photocatalyst for CIP degradation was illustrated in a Z-scheme fashion. In this schema, MXene acted as the electron mediator, expediting the swift transfer of e^−^ from the CB of g-C_3_N_4_ to the VB of BP, where e^−^–h^+^ recombination took place. The e^−^ thus accumulated in the CB of BP and reduced the dissolved O_2_, yielding •O_2_^−^, while the h^+^ in the VB of g-C_3_N_4_ either interacted directly with CIP or generated ^1^O_2_ by reacting with the dissolved O_2_. Furthermore, upon five successive reuses, the CXB maintained a stable degradation efficiency for CIP at levels exceeding 96%, with no apparent alterations in its components observed. This affirmation underscores the potential of anisotropic 2D layered structure, excellent electrical conductivity, and distinctive band structure, establishing MXene and BPNS as promising materials for applications in photocatalysis.

**Figure 8:**
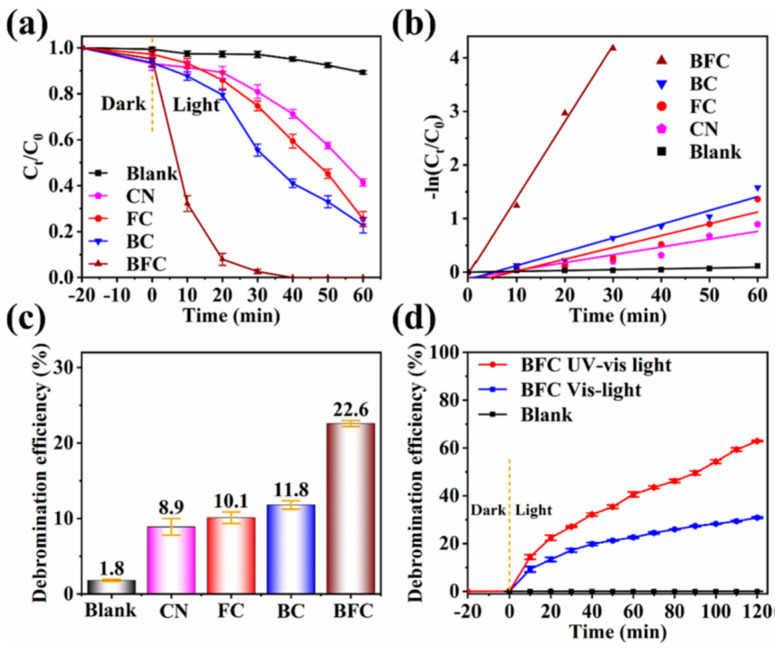
(**a**) Removal, (**b**) rate constant, and (**c**) debromination efficiency of TBBPA over different samples in 60 min under visible light irradiation, (**d**) debromination efficiency of the BFC under visible and UV–vis light irradiation. Blank: pure photolysis. Copyright Year 2023, *Separation and Purification Technology* © Elsevier Pvt Ltd., Amsterdam, The Netherlands.

In recent years, g-C_3_N_4_, a metal-free semiconductor, has garnered substantial attention for its utilization in the elimination of organic pollutants, primarily due to its exceptional and distinctive properties [101]. For instance, its outstanding responsiveness to visible light, non-toxic nature, remarkable stability, adjustable electronic structure, and straightforward synthesis methods make it a compelling choice. Recently, a highly efficient ternary Z-scheme visible-light-driven photocatalyst, denoted as g-C_3_N_4_/BP/MoS_2_ (CBM), was synthesized using a hydrothermal calcination approach [102]. In particular, the well-designed ternary Z-scheme configuration not only enhanced the ability to harness visible light but also expedited the separation and migration of photogenerated carriers. Moreover, the introduction of nitrogen vacancies during the preparation process served to effectively suppress the recombination of photoinduced e^−^–h^+^ pairs. Consequently, CBM demonstrated an exceptionally high degradation efficiency, exceeding 99%, for CIP within 60 min under visible-light irradiation. The experimental results indicated that h^+^ and •O_2_^−^ represented the predominant species responsible for the swift degradation of CIP, effectively breaking down the piperazine and quinolone ring structures. This outcome presented a straightforward approach for creating vacancy-enhanced Z-scheme photocatalysts tailored for the highly efficient degradation of antibiotics in water, particularly under visible-light conditions. What is more, to enhance the photocatalytic performance of pristine g-C_3_N_4_. CBM2 demonstrated marginal reduction in photocatalytic activity following five cyclic experiments, underscoring its potential as a recyclable photocatalyst with practical applicability in water purification. Liu et al. designed and synthesized a p-n/Z-scheme dual heterojunction photocatalyst, denoted as BPNS/FeSe_2_/g-C_3_N_4_ (BFC) [103]. In contrast to conventional heterojunction catalysts, BFC exhibited the dual advantages of enhancing the efficient separation of photogenerated carriers while maintaining a higher redox potential. This multifaceted improvement was achieved through the synergistic effects of creating a built-in electric field in the p–n heterojunction and optimizing the bandgap structure via the Z-scheme heterojunction configuration. These advantages propelled BFC to attain a remarkable 100% Tetrabromobisphenol A (TBBPA) degradation efficiency within 40 min and a substantial 22.6% debromination efficiency in 60 min under visible-light irradiation (Figure 8). What is more, after 10 consecutive cycles of reuse, the BFC exhibited sustained and stable photocatalytic activity, maintaining a consistent level of degradation efficiency.

#### 4.1.4. S-Scheme Heterojunction

**Figure 9:**
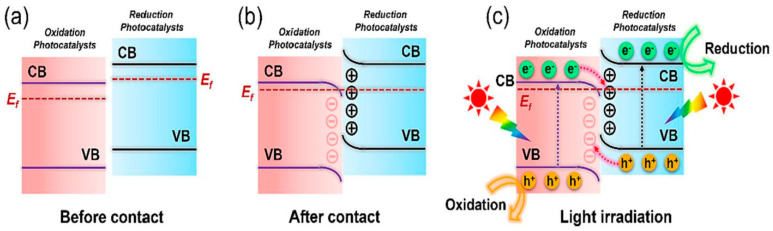
Charge transfer pathway in an S-scheme heterojunction photocatalyst: (**a**) before contact, (**b**) after contact, and (**c**) photogenerated charge carrier transfer under light irradiation. Copyright Year 2023, *Separation and Purification Technology* © Elsevier Pvt Ltd., Amsterdam, The Netherlands.

A recent advancement in the field introduced a novel heterojunction concept known as the S-scheme alignment, which builds upon the principles of the Z-scheme heterojunction [104,105]. Illustrated in Figure 9a, the S-scheme heterojunction consists of two distinct photocatalysts: a reduction photocatalyst (RP) and an oxidation photocatalyst (OP). Notably, the RP exhibits higher CB and VB positions and a smaller work function compared to OP. When RP and OP come into close contact, e^−^ from RP, characterized by a higher Fermi level (E_f_), migrate toward OP with a lower E_f_. Consequently, the RP side loses e^−^ and acquires a positive charge, while the OP side gains e^−^ and becomes negatively charged. Simultaneously, their E_f_ align, leading to band bending in an upward or downward direction at the RP–OP interface (Figure 9b). This configuration generates an internal electric field directed from RP to OP, compelling e^−^ in the CB of OP and h^+^ in the VB of RP to recombine at the contact interface under light irradiation (Figure 9c). As a result, this mechanism achieves effective spatial separation of strongly reductive e^−^ in the CB of RP and potent oxidative h^+^ in the VB of OP, enabling their participation in photocatalytic redox reactions.

**Figure 10:**
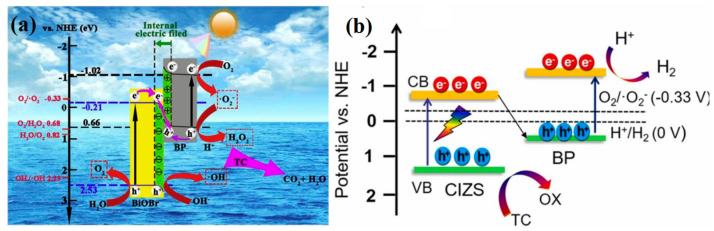
(**a**) S-scheme photocatalytic charge transfer and reaction mechanism between BiOBr and BP. Copyright Year 2020, *Journal of Hazardous Materials* © Elsevier Pvt Ltd., Amsterdam, The Netherlands. (**b**) S-scheme photocatalytic charge transfer and reaction mechanism of BP/CIZS. Copyright Year 2022, *Journal of Alloys and Compounds* © Elsevier Pvt Ltd., Amsterdam, The Netherlands.

Recent research has unveiled the fabrication of an S-scheme heterojunction involving BP and BiOBr using a straightforward approach that combined liquid-phase ultrasound with a solvothermal method [106]. In this configuration, BP served as a reduction photocatalyst, while BiOBr acted as an oxidation photocatalyst. Notably, a difference in work function between BP and BiOBr (Figure 10a) indicated the potential for charge transfer at their contacted interface. According to the S-scheme charge transfer mechanism, unproductive e^−^ in the CB of BiOBr and h^+^ in the VB of BP recombined, whereas the efficient e^−^ and h^+^ in the CB of BP and VB of BiOBr remained available to actively participate in photocatalytic reactions. Consequently, the resulting BP/BiOBr photocatalyst demonstrated an amplified capacity for visible-light-driven degradation of TC and excelled in photocatalytic O_2_ production. The degradation rate exhibited a marginal decrease during the cycling process, albeit not conspicuously. Following four recycling iterations, the degradation rate consistently surpassed 80%.

Wang et al. employed a hydrothermal method to synthesize an S-scheme heterojunction featuring BP/CuInZnS (CIZS) with Zn-P bonds [107]. This innovative approach significantly enhanced the photocatalytic activity for H_2_ production, resulting in an impressive five-fold increase to 1921.2 μmol/g (640.4 μmol/h/g), accompanied by a noTable 82% degradation of TC. According to the experimental results, it was observed that a robust S-scheme heterojunction was established within the BP/CIZS system. This unique configuration preserved the exceptional redox capacity of e^−^–h^+^ pairs by ensuring effective carrier separation. Furthermore, the introduction of the innovative Zn-P bonds served as a conduit for charge transport and catalytic reaction centers, thereby enhancing the photocatalytic performance (Figure 10b). The BP/CIZS composite demonstrated robust photocatalytic stability, with no discernible alterations in activity or crystal structure observed even after five cycles.

Presently, there is a growing interest in BP-based Z-scheme and S-scheme heterojunctions, primarily due to their effective spatial separation of e^−^–h^+^ pairs and substantial redox capabilities. Nevertheless, it is imperative to conduct in-depth investigations into the associated charge transfer mechanisms. This necessitates the comprehensive utilization of advanced characterization methodologies, including spatial-resolved surface photovoltage spectroscopy and ex-situ/in-situ irradiated X-ray photoelectron spectroscopy (XPS), to gain a more profound understanding of these intricate processes.

### 4.2. Hybrids and Doped

**Figure 11:**
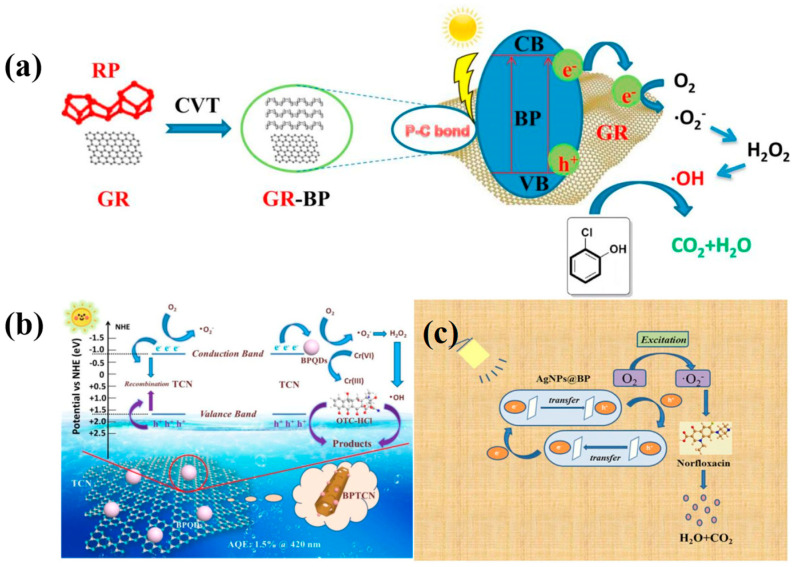
(**a**) The plausible mechanism of BP/GR hybrid for the degradation of 2-CP. Copyright Year 2019, *Environmental Pollution* © Elsevier Pvt Ltd., Amsterdam, The Netherlands. (**b**) Possible photocatalytic mechanism scheme of the carriers separation process on the interface of BPTCN under visible light irradiation. Copyright Year 2020, *Applied Catalysis B: Environmental* © Elsevier Pvt Ltd., Amsterdam, The Netherlands. (**c**) Possible photocatalytic mechanism of the carriers separation process on the interface of AgNPs@BP nanocomposite under near-infrared light irradiation. Copyright Year 2020, *Solid State Sciences* © Elsevier Pvt Ltd., Amsterdam, The Netherlands.

GR has recently been integrated with BP to enhance and stabilize BP’s photocatalytic performance. For example, Zhang et al. employed a one-pot CVT method to synthesize a GR-BP hybrid photocatalyst [108]. The incorporation of GR led to improved charge separation and transfer within the GR–BP hybrid catalyst. Consequently, the GR–BP composite exhibited exceptional photocatalytic activity in the degradation of 2-chlorophenol (Figure 11a). What is more, P–C bonds formed in the synthesized GR–BP hybrid, leading to a reduction in available reactive sites for the incorporation of oxygen atoms into the BP. Even after 15 d, no significant oxidation was observed. In addition, Wang et al. successfully synthesized 0D/1D mixed-dimensional BP/tubular g-C_3_N_4_ (TCN) nanohybrids by employing an ice-assisted ultrasonic method to load BPQDs with an approximate size of 3.32 nm onto TCN structures with diameters ranging from approximately 6 to 8 μm [109]. The resulting BP-TCN nanohybrids demonstrated enhanced photocatalytic performance in the removal of diverse organic pollutants, including oxytetracycline hydrochloride (OTC-HCl), TC, and rhodamine B (RhB), as well as in the reduction of Cr(VI), compared to CN and TCN. Significantly, under visible-light irradiation, the BP-TCN nanohybrids achieved an 81.05% degradation rate for OTC-HCl in simulated wastewater, with an apparent quantum efficiency (AQE) at λ = 420 nm registering at 0.15%. In this configuration, the incorporation of BPQDs onto the surface of TCN leaded to reduced recombination of space charge carriers, thereby enabling a greater number of carriers to participate in the degradation of OTC-HCl (Figure 11b). Conversely, BPQDs, serving as e^−^ mediators, were homogeneously deposited on the surface of TCN, resulting in a distinctive 0D/1D structure characterized by interfacial P−C bonds. This configuration effectively hindered carrier recombination. The intimate interaction established by P−C bonds between BPQDs and TCN facilitated cyclic photostability and electron transfer. In addition, Chen et al. successfully synthesized a novel nanocomposite, AgNPs@BP, through a process involving liquid stripping and centrifugation [110]. The photocatalytic performance of the designed samples was assessed based on the photo-induced degradation of norfloxacin under near-infrared light irradiation. The results demonstrated that under optimal conditions, approximately 85.0% of norfloxacin (NOR) was rapidly degraded within 90 min. Moreover, the AgNPs@BP catalyst exhibited excellent recyclability, retaining catalytic activity over five cycles without significant reduction. The enhanced photocatalytic activity of the AgNPs@BP nanocomposite was attributed to the sensitization of BPNS, enabling the full harvest of near-infrared light and achieving high e^−^–h^+^ separation efficiency (Figure 11c).

Here, Table 3 shows the reaction conditions and efficiency degradation of representative BP-based composite photocatalysts for ECs.

## 5. Conclusions and Perspectives

In recent years, the utilization of BP with various dimensional structures has emerged as a highly promising avenue in the realm of nonmetallic semiconductor photocatalytic materials, primarily due to its distinctive optical and electrical properties. Nevertheless, the intrinsic challenge of rapid recombination of photoinduced carriers in pure BP significantly undermines its overall photocatalytic efficacy. Addressing this, the establishment of a heterojunction interface between BP and other catalysts proves to be an effective strategy for suppressing surface e^−^–h^+^ recombination. Consequently, this article provides a comprehensive overview of the principles guiding the design of BP-based materials and elucidates the mechanisms underpinning the separation and migration of photogenerated carriers in these structures. Furthermore, it compiles the recent advancements in employing BP-based catalysts for photocatalytic ECs removal.

Although significant progresses have undeniably been achieved, the development of high-activity BP-based photocatalysts, suitable for applications in the photocatalytic ECs degradation, continues to present several notable challenges. Concurrently, a series of yet-unanswered questions pertaining to the underlying reaction processes and mechanisms within BP-based heterojunction photocatalytic systems demand comprehensive and in-depth exploration. Building upon the elucidation provided earlier, this article underscores the imperative need for intensified efforts in the following pivotal domains.

(1) To date, a diverse array of 2D nanomaterials, including GR, inorganic hexagonal boron nitride (h-BN), transition metal dichalcogenides (TMDs), and MXenes, as well as g-C_3_N_4_ and covalent organic frameworks (COFs), have been developed as catalysts for a broad spectrum of applications. Beyond the inherent advantages of 2D materials, BPNS exhibited a narrow band gap in the visible region, layer-dependent optical properties, high carrier mobility, and abundant lone-pairs for metal ion anchoring, rendering it a valuable candidate in catalytic fields. For instance, compared with BP, GR has found applications in various fields, including electrical and optical devices, owing to its exceptional carrier mobility, remarkable thermal conductivity, and optical transparency. Nevertheless, its intrinsic zero bandgap property disqualifies it as a proficient photocatalyst since it cannot be photoexcited to generate charge carriers. Conversely, TMDs, another extensively explored 2D crystal, exhibit tunable bandgap energies but suffer from low charge mobility, thereby limiting their suitability as ideal photocatalysts. Nevertheless, BP confronted challenges of instability in ambient environments due to chemical degradation, constituting the primary impediment to its prospective utilization in electronic devices, photocatalysis, and other scientific domains. Furthermore, the large-scale production of few-layer stable BP imposed additional constraints on its applications. Although the Earth’s crust contains abundant phosphorus, the production cost of stable few-layer BP is heightened due to the more stringent conditions required for its preparation compared to other 2D materials. Therefore, the exploration of large-scale production of stable, scalable, and cost-effective few-layer BP is particularly crucial.
(2) The integration of machine learning stands as a promising avenue for guiding the production of high-activity BP-based catalysts for ECs degradation. Currently, machine learning has emerged as a prominent and efficacious research methodology within the realm of photocatalysis. It enables the targeted prediction and selection of photocatalysts possessing requisite properties from extensive, pre-established databases. These encompass critical parameters such as the catalysts’ band structures, work functions, and interfacial interactions of composites, as well as the energy fluctuations associated with surface redox reactions.
(3) Researchers can extend the application of BP-based materials to a wider range of photocatalytic reactions. While the application of BP-based photocatalysts has primarily focused on water splitting for H_2_ generation, there has been limited research on their use in the photocatalytic degradation of ECs. Therefore, it is essential to rationally design BP-based photocatalysts and apply them in the field of photocatalytic degradation of ECs. Investigating the corresponding photocatalytic mechanisms is equally imperative. What is more, the mineralization efficiency of BP-based photocatalysts still requires further enhancement. Previous studies have demonstrated a high photocatalytic degradation efficiency for ECs. However, TOC experiments indicated a certain reduction in mineralization efficiency compared to degradation efficiency. Therefore, further research is needed to investigate the mineralization efficiency of BP-based photocatalytic materials for ECs.
(4) To gain deeper insights into the photocatalytic mechanism, it is imperative to employ advanced characterization techniques and essential theoretical calculations. In addition to conventional methods such as electron spin resonance and experiments for capturing active species, a comprehensive understanding of the catalytic reaction processes in BP-based materials can be achieved through various in situ characterizations including XPS, Fourier-transform infrared (FT-IR), and Raman spectroscopy. These techniques can provide detailed insights into the photocatalytic mechanism. Furthermore, femtosecond time-resolved transient absorption spectroscopy and photoirradiated Kelvin probe measurements are invaluable tools for directly examining the transfer processes of photoinduced charge carriers. Additionally, rational density functional theory (DFT) computations, involving the determination of the lowest-energy structure and local density of states (LDOS), enable a theoretical exploration of the enhanced photocatalytic mechanisms exhibited by BP-based photocatalysts at molecular and atomic levels.

## Figures and Tables

**Table 1:** Different preparation methods of bulk BP.

Method	Precursor	Experimental Condition	Years	References
High-temperature and high-pressure method	WP	200 °C 1.2 GPa	1914	[43]
	RP	heated to 550 °C, melted at around 900 °C, cooled to 600 °C, 1 GPa	1982	[44]
Mercury catalysis and liquid bismuth recrystallization methods	WP	mercury catalyst 360 to 410 °C, 3 d	1955	[45]
	WP	bismuth catalyst 400 °C, 20 h	1981	[46]
Ball milling method	RP	700 r/min, 2 h	2018	[47]
CVT method	RP	Au, Sn, SnI_4_ 600 °C, 5 to 10 d	2007	[50]
	RP	Sn, SnI_4_ 650 °C, cooled to 480 °C	2015	[51]
	RP	Sn, I_2_ 600 °C, cooled to 490–120 °C	2015	[52]

**Table 2:** Different preparation methods of BPNS.

Method	Precursor	Experimental Condition	Years	References
Mechanical exfoliation method	BP	SiO_2_ grid-cutting technology	2014	[57]
	BP	Ball milling technique	2017	[58]
Liquid-phase exfoliation method	BP	Ultrasonic treatment and centrifuge with NMP	2014	[62]
	BP	Ultrasonic treatment and centrifuge with NaOH and NMP	2015	[63]
	BP	Ultrasonic treatment and centrifuge with organic solvent	2016	[64,65]
Electrochemical electrode stripping method	BP	Electrochemical electrode stripping With TBA•PF6	2018	[66]
	BP	Electrochemical electrode stripping With TBA•HSO_4_	2019	[67]
CVD method	RP	CVD method	2016	[77]
	RP	in situ CVD method	2016	[78]
Pulsed laser method	RP	Laser pulse method at 150 °C	2015	[70]
	RP	Laser pulse method at 700 °C and 1.5 GPa	2018	[80]

**Table 3:** Reaction conditions and efficiency degradation of representative BP-based composite photocatalysts for ECs.

Photocatalyst	Photocatalyst Type	Photocatalyst Mass (mg)	ECs	Initial Concentration (mg/L)	Light Source	Removal (%)	Rate Constant (min^−1^)	References
BPNS-BiOBr	Type I	50	CIP	10	300 W Xenon lamp, >420 nm	98.2	0.0245	[82]
BP-g-C_3_N_4_	Type I	10	IDM	5	300 W Xenon lamp, >400 nm	99.2	0.1600	[84]
BP/CN	Type I	5	HTC	5	300 W Xenon lamp, >400 nm	99.2	-	[85]
BPQDs/ATP	Type II	50	TPA	50	300 W Xenon lamp, 200–780 nm	90.0	-	[86]
BiOBr/UCN/BPQDs	Type II	250	TC	30	300 W Xenon lamp >420 nm	92.0	0.0410	[87]
BP/RP-g-C_3_N_4_/SiO_2_	Type II	5	OFL	10	350 W Xenon lamp >420 nm	85.3	0.0370	[88]
BP/CeO_2_	Z-scheme	50	BPA	50	300 W Xenon lamp, 200–780 nm	82.3	-	[89]
F-BP/BiOI	Z-scheme	25	TC	10	300 W Xenon lamp >420 nm	90.0	0.0767	[92]
BPQDs/BiOBr	Z-scheme	-	TC	20	400 W metal halide lamp, >420 nm	97.5	0.4603	[93]
Bi_2_WO_6_/g-C_3_N_4_/BPQDs	Z-scheme	40	BPA	20	300 W Xenon lamp >380 nm	95.6	0.0439	[94]
g-C_3_N_4_/Ti_3_C_2_ MXene/BP	Z-scheme	20	CIP	20	300 W Xenon lamp >420 nm	99.0	0.0480	[100]
g-C_3_N_4_/BP/MoS_2_	Z-scheme	20	CIP	20	300 W Xenon lamp >420 nm	99.0	0.0600	[102]
BPNS/FeSe_2_/g-C_3_N_4_	Z-scheme	20	TBBPA	10	300 W Xenon lamp 380–780 nm	100.0	0.1430	[103]
BP/BiOBr	S-scheme	100	TC	50	300 W Xenon lamp 420–780 nm	85.0	0.0210	[106]
BP/CIZS	S-scheme	35	TC	200	300 W Xenon lamp 420–780 nm	82.0	-	[107]
GR-BP	Hybrid	50	2-CP	10	300 W Xenon lamp >420 nm	87.1	-	[108]
BP-TCN	Hybrid	30	OTC-HCl	10	300 W Xenon lamp >420 nm	81.1	0.0276	[109]
AgNPs@BP	Doped	-	NOR	15	300 W Xenon lamp 880 nm	84.8	-	[110]

## Data Availability

Data will be given upon request.
